# Lifestyle Segmentation to Explain the Online Health Information–Seeking Behavior of Older Adults: Representative Telephone Survey

**DOI:** 10.2196/15099

**Published:** 2020-06-12

**Authors:** Winja Weber, Anne Reinhardt, Constanze Rossmann

**Affiliations:** 1 University of Erfurt Erfurt Germany

**Keywords:** older adults, online health information seeking, lifestyle, segmentation, cluster analysis

## Abstract

**Background:**

As a result of demographic changes, the number of people aged 60 years and older has been increasing steadily. Therefore, older adults have become more important as a target group for health communication efforts. Various studies show that online health information sources have gained importance among younger adults, but we know little about the health-related internet use of senior citizens in general and in particular about the variables explaining their online health-related information–seeking behavior. Media use studies indicate that in addition to sociodemographic variables, lifestyle factors might play a role in this context.

**Objective:**

The aim of this study was to examine older people’s health-related internet use. Our study focused on the explanatory potential of lifestyle types over and above sociodemographic variables to predict older adults’ internet use for health information.

**Methods:**

A telephone survey was conducted with a random sample of German adults aged 60 years and older (n=701) that was quota-allocated by gender, age, educational status, and degree of urbanity of their place of residence.

**Results:**

The results revealed that participants used the internet infrequently (mean 1.82 [SD 1.07]), and medical personnel (mean 2.89 [SD 1.11]), family and friends (mean 2.86 [SD 1.21]), and health brochures (mean 2.85 [SD 1.21]) were their main sources of health information. A hierarchical cluster analysis based on values, interests, and leisure time activities revealed three different lifestyle types for adults aged over 60 years: the Sociable Adventurer, the Average Family Person, and the Uninterested Inactive. After adding these types as second-step predictors in a hierarchical regression model with sociodemographic variables (step 1), the explained variance increased significantly (*R*^2^=.02, *P*=.001), indicating that the Average Family Person and the Sociable Adventurer use the internet more often for health information than the Uninterested Inactive, over and above their sociodemographic attributes.

**Conclusions:**

Our findings indicate that the internet still plays only a minor role in the health information–seeking behavior of older German adults. Nevertheless, there are subgroups including younger, more active, down-to-earth and family-oriented males that may be reached with online health information. Our findings suggest that lifestyle types should be taken into account when predicting health-related internet use behavior.

## Introduction

### Background

The world population is aging; in 2017, at least 962 million people worldwide were 60 years old and older. Researchers expect a doubling of this number by 2050 [1]. The reasons for this tendency are an increase in life expectancy and a decline in birthrates. This demographic change implies multiple challenges for society, especially for the health care system and health care communicators because aging is linked with several health-related restrictions: research predicts a sharp increase in age-related diseases [2,3], which will lead to a greater need for medical care [4]. For this reason, it is important to effectively inform older adults about health issues, help them manage (chronic) diseases, and promote a healthy lifestyle and, in doing so, relieve the health care system in the long run. In order to guarantee successful communication, it is important to understand what sources of health information seniors use and which predictors best explain their health information–seeking behavior.

In general, the internet has become increasingly important as a source of health information. Research indicates that there has been a great increase in internet use for health information among several target groups [5-8]. Several studies even proclaim the internet to be the most important source of health information nowadays [7,8]. However, other studies show that sources such as interpersonal communication with health care providers or traditional media channels are still more important than the internet [9]. Among older adults, both data on general internet use [10] and data on online health information seeking [9,11] indicate that the internet does not play a major role as a source of health information among older adults.

Given the rapid evolution in this area and the contradictory view on the role of the internet for older adults, we sought to examine the role of the internet as a source of health information for older adults in Germany and, in particular, identify which attributes best explain their online health information–seeking behavior. Research findings indicate that sociodemographic variables are strong predictors of online health information behavior [11], but they do not explain all of it. Thus, explaining the use of the internet for health information using only sociodemographic variables reflects only a part of the picture. Studies in the context of consumer research have considered lifestyle attributes in order to better understand and explain user behavior [12]. Accordingly, online health information–seeking behavior might be influenced by lifestyle factors and values.

Thus, our study focused on the role of lifestyle variables to explain older adults’ online health information behavior. In our article, we focus on adults aged 60 years and older because in the field of gerontology and consumer research it is a common practice to subdivide old age into those aged under 60 years and those aged 60 years or older [13-15]. Because there has been no research showing the impact of lifestyles as predictors of older adults’ online health information behavior, we investigated in a representative survey (1) the role of the internet for health information–seeking behavior among German adults ages 60 years or older and (2) to what extent lifestyle types contribute to the model over and above sociodemographic variables.

### Health Information–Seeking Behavior of Older Adults

According to the Health Information National Trends Survey, in 2014 at least 45% of Americans stated that the internet was their primary source of health information, followed by health care professionals, traditional media, and family and friends [7]. In Germany, representative data showed a similar picture— for instance, in 2013 40% of Germans used the internet for health information [16,17].

However, for adults aged 60 years and above, findings present a different picture. People in this age group predominantly consult health care professionals to obtain health-related information [18,19]. Moreover, they typically use traditional media such as print media, radio, TV, and material from health insurance funds or pharmacies for health-related information [17,20,21]. Although internet use for health-related information among older adults has been steadily increasing over the past years, it still seems to play a minor role. In 2013, just 16% of German adults 60 years and older indicated the use of the internet for health purposes [17]. In America, the number is slightly higher, with 29% of people 65 years and older using the internet to obtain health information [5]. Due to increasing internet use in general during the past several years and a lack of data about the current status quo, we asked:

- RQ1: Which sources of health information do older adults use in Germany and what role does the internet play in this context?

### Factors Explaining Online Health Information–Seeking Behavior of Older Adults

Online health information seeking can be determined by many different factors. A representative study in Europe examined age as one of the strongest predictors of online health information behavior [11]. With increasing age, the number of people who use the internet to seek health-related information decreases [9,18]. Those differences between younger and older people might be a result of psychological and social aging processes. Thus, research found that older people have less technological control and self-efficacy in addition to fewer skills for adapting to new technologies [22]. This lack of confidence can reduce the intention to use online health information [23]. Additionally, adults do not trust the internet as a source of health information [9] and have difficulties in coping with the information overload they find when seeking information online [24]. Other age-related factors that determine internet use are people’s cognitive capability (such as memory loss) and physical conditions (such as sight loss) [25]. Furthermore, people who are generally curious about life in particular search more actively for health information online [26].

In addition to age, research findings indicate that well-educated people who live in an urban area are more likely to use the internet for health-related purposes than those who are less educated and live in a rural area [11,16,27]. Research focusing on the influence of gender-specific online health information–seeking behavior demonstrates heterogeneous findings: while men generally use the internet more often, women are more interested in health information [28]. Accordingly, some researchers found more frequent health-related use of the internet for females [29,30], while others indicate that the typical online health information seeker is male [31].

Another discrepancy in research findings can be found with respect to individuals’ health status. On the one hand, studies indicate that people with poorer health are more likely to search for health information online than people with a good health status [11,26]. On the other hand, studies suggest that adults with a good health status use online health information sources more frequently than those with a poorer self-reported health status [32]. Given the inconclusive picture, we ask the following question:

- RQ2: Which factors (age, education, gender, place of residence, health status) explain older adults’ online health information–seeking behavior?

### Lifestyle Segmentation to Explain Online Health Information–Seeking Behavior

Differences in (consumer) behavior cannot only be explained by demographic variables but rather are influenced by people’s general lifestyles as well [33]. Lifestyles can be defined as “patterns of action that differentiate people.... Lifestyles therefore help to make sense of...what people do, and why they do it, and what doing means to others” [34]. Lifestyle segmentation is a concept derived from market or audience research [12] that aims to cluster people with similar characteristics or values. It has been used primarily in the marketing sector [35] but also in the context of health campaign planning [12]. In marketing and consumer research literature, the term lifestyle is often used synonymously with psychographics [36]. However, psychographics mainly focus on personality traits whereas lifestyles mainly focus on needs and values closely associated with behavior [37]. Because of this, we decided to refer to the lifestyle concept instead of psychographics in this study.

In the context of lifestyles, audience segmentation can be seen as “classification schemes...[that] are based in overarching models and assumptions about...lifestyles and values and not on variables that predict a specific behavior” [38]. Lifestyle is primarily defined by activities, interests, and opinions [33,39-41]. Accordingly, activities (such as work or hobbies), interests (such as family, home, media), and beliefs (such as social issues, politics, culture) are typically measured in order to identify lifestyle types [42]. In addition, researchers often relate lifestyles to values [43] because they are a part of individuals’ views of the world and thus form a major factor in people’s character and behavior [33,44].

In the context of consumer research and audience segmentation, previous studies have already addressed lifestyle attributes to explain consumer behavior [33]. In the specific case of media use, lifestyle segmentation has been used to understand habits such as daily newspaper use [45], adolescents’ media use [46], adoption and use of pagers and mobile phones [41], older adults’ participation in online communities [47], and attitudes toward internet advertisements [48]. While these studies found differences in the behavior between the examined lifestyle types, another study investigated whether lifestyles have the potential to explain online news usage [49]. The authors found that lifestyles did not predict the adoption of online news in general. However, the enjoyment of interactive capability of online news was predicted most strongly by lifestyles in the group of adopters [49].

Most of the aforementioned studies included internet use either as a cluster-forming variable rather than a dependent variable [50,51] or studied differences within the clusters and not the explanatory potential of lifestyles [41,45-48]. Additionally, there is an ongoing debate about whether lifestyles, compared with demographics, are suitable to explain media use patterns. Skeptical researchers suggest that lifestyles are already included in demographic variables [52]. Hence, one study compared the influence of demographics and psychographics to explain the consumption of different forms of media use [53]. It found that the contribution of demographics and psychographics varied across the different behaviors: while psychographics played no role beyond the demographic explanation in outcomes such as genre preferences, the prediction of TV consumption benefited from adding lifestyle variables. Therefore, the authors of this study recommend integrating both approaches to explain and understand behavior [53].

The role of lifestyle variables becomes particularly important when focusing on older adults: several studies show that age itself is not the main predictor for behavior among older people [47,54,55]. A reason might be that aging is a very individualistic process: rather than aging chronologically, people age very differentially [54,56]. How old someone feels is directly associated with their mindset (eg, values) and activity—thus, lifestyle variables play a major role in explaining behavior [54]. For instance, a 65-year-old person can have a very inactive lifestyle and may wish to stay at home most of the time, while a 75-year-old person can be still very sporty and interested in discovering new things. This example might make clear why older people are often described as the most heterogeneous audience [47]. Lifestyle types have already been successfully used to segment this heterogeneous target group into homogenous subgroups to get a better understanding of the so-called gray market [50,51,57-59], but there is no research examining the impact of lifestyle segmentation on the online health information–seeking behavior older adults.

In sum, demographic variables such as age, education, gender, and health status have an influence on online health information–seeking behavior. However, in many cases lifestyle types can go beyond demographics in explaining behaviors. Nevertheless, there has been no research examining the impact of lifestyle segmentation on the online health information–seeking behavior in general and especially of older adults. This led us to ask the following:

- RQ3: Which lifestyle types can be identified in the age group 60 years and older based on general values, leisure values, leisure activities, and interests?
- RQ4: Do lifestyle types offer added value to explain older adults’ online health information–seeking behavior beyond demographics?

## Methods

### Recruitment

To answer our research questions, we conducted a telephone survey with 701 adults aged 60 years and older recruited by the Institute for Applied Marketing and Communication Research GmbH. As the study involved human participants, an institutional review board approval was obtained by the advisory board on ethical issues of the University of Erfurt (No. 17/05/29). All participants gave their informed consent to use and share their data for scientific purposes without disclosure of their identity. The respondents were selected randomly and quota-allocated by age (60-64 years, 65-74 years, and >75 years), rural/urban living areas (<10,000 residents, >10,000 residents), gender (male, female), and education (low, high). The survey was conducted as part of the formative research for the strategic development of a vaccination campaign and therefore also included health-related questions not relevant here. For this reason, the interview duration was about 30 minutes.

About half (15,967/31,419, 50.82%) of the total sample did not fit into the quota or were not reached. In addition, 46.57% (14,633/31,419) of the respondents refused to participate. Since the response rate was low (701/31,419 [2.23% of the gross sample]), the telephone survey did not fully reach the targeted quotas. Because of this, a weighting factor was applied in the following analyses, except in the factor analysis and cluster analysis—SPSS Statistics (IBM Corporation) does not allow the use of weighting in those data analytic procedures.

### Measurement

#### Dependent Variables

Participants’ health information–seeking behavior was measured using sources of health information with 11 items—internet use, health personnel, brochures, family and friends, conventional media (TV, radio, newspapers), apps, pharmacy, books, other patients, health insurance, and health workshops—on a 5-point scale (How often do you use the following sources of health information in general? 1 = never to 5 = very often) [60,61].

#### Lifestyle Variables

As a central determinant of lifestyle types, we measured general values and life goals with 11 items on a 5-point scale (1 = not at all important to 5 = very important): law and order, safety, success, creativity, political commitment, amusement, conformity, friends, environment, peace and harmony, and religion.

In addition, we assessed participants’ leisure values with 3 items (experience adventures, meet interesting people, and do something new/crazy) on a 5-point scale (1 = not at all important to 5 = very important).

To examine the recreational activity of our participants, we asked how often they engage in the following leisure time activities during their free time (eg, at weekends, in the afternoon): using a computer, participating in sports, going to the theater/opera, visiting a museum, eating out in a restaurant, having guests visit them at home, reading books, watching TV, gardening, doing handicraft work, going for a walk, baking/cooking, listening to music, and spending time with their family. The frequency was measured on a 5-point scale (1 = never to 5 = very often).

Participants’ personal interests were assessed on a 5-point scale (1 = not interested in to 5 = very interested in) via 11 items: politics, economics/law, sports, health, arts/literature, science, children/upbringing, partnership/family, traveling/vacation, work, and house/garden.

#### Further Predictor Variables

Participants’ subjective health status was measured on a 5-point scale (1 = very bad to 5 = very good) with the question: How would you describe your actual state of health? (mean 3.06 [SD 0.80]) [62].

Finally, we assessed age, gender (0 = male, 1 = female), educational level (0 = low, including no educational qualifications, secondary modern school until class 9, or junior high; 1 = high, including university-entrance diploma, university degree, or higher), and the urbanity of place of residence (0 = under 10,000 residents, 1 = more than 10,000 residents).

### Power Analysis

A power analysis determined the sample size for inferential statistics (independent *t* test: power=.80, α=.05, effect size *d*=0.2) and was rounded up to the nearest hundred [63].

## Results

### User Statistics

After weighting, the average age of participants was 71.6 (SD 7.3) years and 55.3% (388/701) were female. Most subjects had lower education levels (590/693, 86.0%) and came from cities with a population of more than 10,000 residents (518/701, 73.9%). Regarding their technical equipment, nearly two-thirds of our sample owned a computer (464/698, 66.2%) and nearly half of them had a smartphone (289/699, 41.4%). Furthermore, 60.7% of the participants (425/697) used the internet at least occasionally with an average duration of 85.2 (SD 109.9) minutes per day. See Multimedia Appendix 1 for descriptive analyses of all variables included in the following main analyses.

### Evaluation Outcomes

#### Health Information Behavior of Older Adults

For our first research question (RQ1), we wanted to know what health information sources older adults use and if the internet plays a role in this context. A descriptive analysis (Table 1) showed that the most commonly used sources of health information were free brochures and pharmacy magazines, followed by interpersonal communication with medical staff, family, and friends, and traditional media sources. The internet was only infrequently used as a source of health information (mean 1.82 [SD 1.08]). Only other patients, health insurances, health workshops, and apps are used less often than the internet to get health information.

**Table 1:** Frequency of older adults’ use of health information sources (n=681-689).

Source	Mean (SD)	95% CI
Health personnel	2.89 (0.04)	2.81-2.98
Family/friends	2.87 (0.40)	2.80-2.95
Brochures	2.85 (0.05)	2.76-2.94
Conventional media	2.70 (0.04)	2.61-2.78
Books	2.01 (0.04)	1.93-2.09
Pharmacy	1.97 (0.04)	1.90-2.04
Internet	1.83 (0.04)	1.78-1.91
Other patients	1.80 (0.04)	1.73-1.87
Health insurance	1.42 (0.03)	1.34-1.47
Health workshops	1.29 (0.03)	1.23-1.34
Apps	1.12 (0.02)	1.08-1.15

#### Lifestyle Types of Older Adults

In order to identify lifestyle types among adults aged 60 years and above (RQ3), we combined factor analyses with a cluster analysis. Because all lifestyle constructs were assessed via numerous items, in a first step we conducted exploratory factor analyses (rotation method: varimax; missing data: listwise deletion) with principal axis factoring in order to group similar variables into dimensions. Items that could not be summed up to one of the factors were treated separately in the cluster analysis.

Four of the 11 general values items could be combined into 2 factors (Kaiser-Meyer-Olkin [KMO]=.539; Multimedia Appendix 2), including the dimensions harmony (high loadings on importance of friends, peace/harmony) and regularity (high loadings on importance of law and order, safety).

The leisure activities were assessed with 14 items, of which 6 could be summed to 3 factors (KMO=.501; Multimedia Appendix 3), including culture (high loadings on going to a theater/opera, visiting museums), home and garden (high loadings on handicraft work, gardening), and technology (high loadings on using a computer, anti-baking/cooking). The leisure values (3 items) were treated separately (in accordance with the underlying scale).

The participants’ personal interests were measured with 11 items that were combined into 2 factors (KMO=.580; Multimedia Appendix 4), including news (high loadings on interest in politics, economics/law) and family (high loadings on interest in children/upbringing, partnership/family, work/education).

In order to identify homogenous groups of cases based on the above-mentioned lifestyle variables, a hierarchical cluster analysis was computed. Ward minimum-variance clustering was performed using the squared Euclidean distance. Prior to the hierarchical cluster analysis, the relevant assumptions of this statistical analysis were tested [64,65]: (1) sample size of n=701 was deemed adequate, (2) statistical outliers were eliminated, (3) there were no missing data, (4) all analyzed variables were z-standardized, and (5) cluster variables were tested for normal distribution (the latter assumption was violated, but the hierarchical cluster analysis is robust to this violation) [66]. At each step, samples were merged into larger clusters to maximize the between-cluster sum of squares. Using this approach, three clusters were identified. They differed significantly in all cluster variables except for the leisure activity domestic work (Table 2).

**Table 2:** Differences in general values and life goals, leisure values, leisure activities, and interests between the identified clusters (n=595).

Variables		Total cohort, mean (SD)	Social Adventurer, mean (SD)	Average Family Person, mean (SD)	Uninterested Inactive, mean (SD)	F score	*P* value
**General values**							
	Regularity	0.02 (0.79)	–0.19 (0.90)^a^	0.02 (0.77)^b^	0.30 (0.52)^c^	13.93	<.001
	Harmony	0.01 (0.61)	0.07 (0.52)^a^	0.03 (0.62)^a^	–0.15 (0.66)^b^	5.05	.01
	Success	0.00 (1.00)	0.34 (0.94)^a^	–0.14 (0.97)^b^	–0.14 (1.02)^b^	14.82	<.001
	Creativity	0.01 (0.98)	0.45 (0.64)^a^	–0.02 (0.90)^b^	–0.54 (1.25)^c^	40.57	<.001
	Political commitment	0.00 (0.99)	0.52 (0.94)^a^	–0.03 (0.97)^b^	–0.65 (0.65)^c^	57.78	<.001
	Amusement	–0.01 (0.99)	0.56 (0.82)^a^	–0.11 (0.96)^b^	–0.56 (0.87)^c^	56.72	<.001
	Conformity	–0.03 (0.96)	0.09 (0.97)^a^	–0.01 (1.01)^a^	–0.23 (0.77)^b^	3.87	.02
	Environment	0.02 (0.99)	0.27 (0.78)^a^	–0.11 (1.10)^b^	–0.03 (0.91)^b^	8.32	<.001
	Religion	–0.01 (1.00)	–0.00 (1.02)^a^	–0.01 (1.01)^a^	0.00 (0.92)^a^	0.01	>.99
**Leisure values**							
	Experience adventures	–0.01 (0.99)	0.74 (1.00)^a^	–0.20 (0.88)^b^	–0.61 (0.50)^c^	100.48	<.001
	Meet interesting people	0.02 (1.00)	0.51 (0.60)^a^	0.03 (0.94)^b^	–0.73 (1.15)^c^	64.23	<.001
	Do something crazy	0.01 (0.99)	0.71 (1.01)^a^	–0.18 (0.87)^b^	–0.52 (0.69)^c^	82.91	<.001
**Leisure activities**							
	Culture	0.01 (0.85)	0.59 (0.79)^a^	–0.03 (0.77)^b^	–0.70 (0.51)^c^	105.06	<.001
	Technology	0.01 (0.63)	0.05 (0.66)^a^	0.03 (0.62)^a^	–0.12 (0.57)^b^	3.08	.047
	Home and garden	0.03 (0.76)	0.10 (0.77)^a^	0.10 (0.75)^a^	–0.24 (0.72)^b^	10.01	<.001
	Participating in sports	0.04 (0.99)	0.60 (0.76)^a^	0.03 (0.96)^b^	–0.75 (0.77)^c^	83.27	<.001
	Eating out in a restaurant	0.01 (0.99)	0.28 (0.98)^a^	0.04 (0.95)^b^	–0.46 (0.95)^c^	20.44	<.001
	Having guests	0.00 (0.98)	0.52 (0.75)^a^	–0.00 (0.90)^b^	–0.74 (1.01)^c^	70.30	<.001
	Reading books	0.01 (1.00)	0.57 (0.71)^a^	0.09 (0.98)^b^	0.01 (0.90)^c^	51.87	<.001
	Watching TV	0.01 (0.98)	–0.12 (1.02)^a^	0.03 (0.96)^a^	–0.75 (0.77)^a^	2.56	.08
	Going for a walk	0.07 (0.96)	0.51 (0.74)^a^	0.00 (0.94)^b^	–0.39 (1.04)^c^	36.02	<.001
	Listening to music	0.01 (0.99)	0.26 (0.88)^a^	0.07 (0.91)^a^	–0.52 (1.13)^b^	24.70	<.001
	Spending time with family	0.03 (0.96)	0.28 (0.88)^a^	0.02 (0.96)^b^	–0.31 (0.97)^c^	13.62	<.001
**Interests**							
	News	0.00 (0.82)	0.16 (0.73)^a^	0.08 (0.77)^a^	–0.46 (0.93)^b^	24.70	<.001
	Family	0.01 (0.85)	0.27 (0.82)^a^	0.11 (0.76)^a^	–0.62 (0.81)^b^	48.21	<.001
	Sports	0.01 (1.00)	0.39 (0.89)^a^	–0.07 (0.99)^b^	–0.32 (1.03)^c^	20.35	<.001
	Health	0.02 (0.98)	0.18 (0.96)^a^	–0.03 (1.01)^b^	–0.09 (0.94)^b^	3.52	.03
	Arts and literature	–0.02 (0.99)	0.55 (0.84)^a^	–0.04 (0.91)^b^	–0.80 (0.86)^c^	81.19	<.001
	Science and technology	0.02 (1.00)	0.46 (0.81)^a^	–0.04 (0.99)^b^	–0.47 (1.04)^c^	32.59	<.001
	Traveling and vacation	0.02 (1.00)	0.46 (0.81)^a^	0.09 (0.91)^b^	–0.82 (0.97)^c^	73.85	<.001
	House and garden	0.02 (0.99)	–0.12 (1.01)^a^	0.20 (0.83)^b^	–0.24 (1.10)^a^	11.31	<.001

^a-c^Cells in a row with different letter superscripts differ with *P*<.05 (Duncan post hoc test).

Cluster 1 consisted of 28.5% (169/595) of the total subjects. This cluster was characterized by participants who were interested in almost all questioned activities. They stated an appreciation for meeting interesting people, doing something crazy, and experiencing adventures. In addition, they liked to have guests, read books, and spend time actively (eg, doing sports, visiting a museum). Furthermore, they attached great importance to a life full of creativity, amusement, and political engagement. Hence, cluster 1 was called the Sociable Adventurer.

The second cluster was the largest group, with 51.7% (308/595) of the total subjects. This group included older adults who put less value on experiencing adventures or doing something crazy. Instead, they appreciated doing domestic work and gardening or spending time with their families. In general, they did not focus on a life full of success or amusement. Therefore, we called this type the Average Family Person.

Cluster 3 was the smallest group (118/595, 19.8%) and significantly different from the others, characterized by participants who had no real interests and did not like to leave their comfort zones (such as having no interest in experiencing adventures or trying something new). These subjects had a great sense for regularity. Cluster 3 was called the Uninterested Inactive. For a more comprehensive overview of the three lifestyle types, we look at their demographics in the next step (Table 3).

**Table 3:** Characteristics of clusters by demographic variables.

Demographics		Sociable Adventurer, n (%)	Average Family Person, n (%)	Uninterested Inactive, n (%)
**Age in years**				
	60-64	48 (28.4)	76 (24.8)	25 (21.2)
	65-69	45 (26.6)	65 (21.2)	13 (11.0)
	70-74	25 (14.8)	55 (17.9)	18 (15.3)
	75+	51 (30.2)	111 (36.2)	62 (52.2)
**Gender^a^**				
	Female	81 (47.9)	126 (41.0)	66 (55.9)
	Male	88 (52.1)	181 (59.0)	52 (44.1)
**Education^a^**				
	Low	67 (40.1)	172 (56.0)	87 (74.4)
	High	100 (59.9)	135 (44.0)	30 (25.6)
**Place of residence^a^**				
	Smaller city	23 (13.5)	94 (30.5)	39 (33.1)
	Middle sized or larger city	147 (86.5)	214 (69.5)	79 (66.9)

^a^Demographic factors differ with *P*<.05 (χ^2^ difference test).

Corresponding to the degree of activity, the oldest participants belonged to cluster 3. This cluster was also characterized by individuals with lower educational status. With regard to the place of residence, it becomes obvious that cluster 1 mainly consisted of people who lived in middle sized or larger cities. Last, cluster 2 can be characterized by a high proportion of men as compared with the other lifestyle types.

#### Factors Influencing Online Health Information–Seeking Behavior

In order to analyze which factors influence online health information seeking among older adults (RQ2 and RQ4), we conducted a 2-step hierarchical regression analysis with internet use for health information as the dependent variable (for an overview of distribution of the outcome variable, see Multimedia Appendices 1 and 5). The independent variables age, gender, educational status, health status, and place of residence were entered in step 1 and the lifestyle types in step 2. Because of the significantly different characterization of cluster 3, the Uninterested Inactive (dummy) served as reference group for the variables the Sociable Adventurer (dummy) and the Average Family Person (dummy). Prior to the hierarchical regression, the relevant assumptions of this statistical analysis were tested. First, the sample size was deemed adequate for the 7 independent variables to be included in the analysis [67]. The assumption of singularity was also met, as the independent variables were not a combination of other independent variables. An examination of correlations revealed that no independent variables were highly correlated. Also, there was no multicollinearity as indicated below (tolerance >0.10, variance inflation factor <10) [68]. Residual and scatter plots indicating the assumptions of normality, linearity, and homoscedasticity were all satisfied [69,70].

Hierarchical regression revealed that at step 1, demographic variables contributed significantly to the regression model (*R*^2^=.114, *F*_5,582_=14.92, *P*<.001). As can be seen in Table 3, age correlated negatively with the frequency of online health information seeking (β=–.236, *P*<.001). Accordingly, younger elderly used the internet more often than older ones as a source of health information. Another significant predictor was participant gender. Specifically, men showed greater online heath information–seeking behavior than woman (β=–.131, *P*=.001). Moreover, educational status (β=.126, *P*=.002) and health status (β=.081, *P*=.04) correlated positively and significantly with online health information use, indicating that highly educated and healthy seniors tend to use online health information more often than less well-educated subjects and those with poorer self-reported health. The place of residence was not significantly associated with health-related use of the internet.

Introducing the lifestyle types in step 2 explained another 2 percentage points of variance (*R*^2^=.132). This change in *R*^2^ was significant (*F*_7,580_=12.57, *P*<.001). Again, the demographic variables age (β=–.208, *P*<.001), gender (β=–.151, *P*<.001), and education (β=.093, *P*=.03) correlated significantly with internet use, whereas the influence of subjects’ health status disappeared. Both lifestyle types influenced online health information significantly; that is, the Sociable Adventurer (β=.186, *P*<.001) and the Average Family Person (β=.160, *P*=.003) used online health information more often than the Uninterested Inactive (Table 4). To examine differences between the Sociable Adventurer and the Average Family Person, we repeated the analysis with changed reference group (Multimedia Appendix 6). We found no significant influence of the Adventurer type when comparing it with the Average Family Person. Therefore, while both the Sociable Adventurer and the Average Family Person use the internet for health information more often than the Uninterested Inactive, there is no significant difference between them.

**Table 4:** Summary of hierarchical multiple regression analysis for variables predicting older adults’ online health information–seeking behavior (n=587).

Predictors		Step 1				Step 2				
		B^a^	SE B^b^	β^c^	*P* value		B	SE B	β	*P* value
**Step 1**										
	Age	–0.033	0.005	–0.236	<.001		–0.029	0.006	–0.208	<.001
	Gender	–0.281	0.085	–0.131	.001		–0.323	0.085	–0.151	<.001
	Education	0.270	0.087	0.126	.002		0.199	0.088	0.093	.03
	Place of residence	0.184	0.096	0.076	.05		0.155	0.096	0.064	.11
	Health status	0.102	0.050	0.081	.04		0.068	0.051	0.054	.18
**Step 2**										
	Sociable Adventurer (dummy)	N/A^d^	N/A	N/A	N/A		0.439	0.132	0.186	.001
	Average Family Person (dummy)	N/A	N/A	N/A	N/A		0.341	0.114	0.16	.003

^a^B: unstandardized regression coefficient.

^b^SE B: standard error for unstandardized regression coefficient.

^c^β: standardized regression coefficient.

^d^N/A: not applicable.

Due to the fact that internet use for health information did not show wide variance in our sample, we verified our results by testing a logistic regression model recoding internet use as a dummy variable (0 = never, 1 = at least infrequently). The tested model was significant (χ^2^_2_=9.853, *P*=.007). Findings indicate the same tendencies as the linear regression model (Multimedia Appendix 7).

## Discussion

### Principal Results and Comparison With Prior Work

Our study examined the relevance of the internet as a source of health information for older adults in Germany and the explanatory potential of lifestyle types over and above sociodemographic variables. Our findings highlight the fact that the internet still has a minor role compared with other sources in the health information–seeking behavior of older adults. Traditional health information sources such as brochures or medical consultations still are their major sources of health information. These findings are in line with current research showing that traditional media and health care workers are the most important health information sources for older adults [19,20].

A hierarchical cluster analysis based on values, interests, and leisure time activities revealed three different lifestyle types: (1) the first type was the Sociable Adventurer; these subjects were the most active with many interests and free time activities; (2) around 50% of the total participants were assigned to the Average Family Person; they preferred to do domestic work and gardening or spend time with their families; and (3) the smallest number of participants belonged to the Uninterested Inactive type; they were characterized by a lack of interests and an increased need for security.

There were several factors that abet the online information–seeking behavior of older adults. In line with current research, participants’ age, education, and gender were, in particular, defining factors [11,27]: younger, highly educated participants used the internet more often for health information seeking than older participants with lower education. Regarding the heterogeneous evidence of the influencing role of gender on online health information–seeking behavior, our study suggests that the over 60-year-old male segment used the internet more often than women. This finding may be explained by the higher internet use of men in general [28] and in the age group of older adults in particular [71]. Furthermore, it seems reasonable that the age differences in internet use arose from cohort effects within the different age segments. A representative study showed that in 2017, Germans aged 50 to 69 years used the internet 98 minutes per day, while older adults (>70 years) used it much less (36 minutes per day) [72]. These numbers suggest that the internet will become an increasingly important health information source for the next generation of older people.

In addition, our study showed that beyond demographic variables, lifestyle types contributed significantly to our model and were relevant factors in predicting online health information–seeking behavior. Specifically, we found active and open-minded older adults (the Sociable Adventurer) as well as down-to-earth and family-oriented participants (the Average Family Person) to be more likely to seek online health information than uninterested, inactive ones (the Uninterested Inactive). This finding is in line with the results of another study indicating that people who are particularly curious and interested in many things use the internet more often for seeking health information [26]. Interestingly, by introducing the lifestyle types to the regression model, the influence of health status on the dependent variable disappeared. An explanation for this finding might be the fact that health status is associated with a person’s lifestyle; lifestyle, in turn, includes many more dimensions (eg, interests, values) and seems to be more appropriate to predict the online health information–seeking behavior of older adults than health status alone.

The results indicate that demographics alone are not adequate to explain the use of internet sources for health information and lifestyles play an important role as well. Although the additional explained variance was rather small at 2%, lifestyle variables do explain significantly more than demographics alone. However, this small effect is in line with other studies that have either found no effect beyond sociodemographic variables (online news usage [49]) or only a very small effect (consuming various forms of media [53]). However, these studies also found that the explanatory potential of lifestyles varies between different behaviors [53] or may become more important if they are used to explain not only general media use but specific patterns within a behavior [49]. In addition, it should be noted that there are currently no other comparative studies, as most studies in this context rely on variance analyses to show differences in media use between different types of lifestyles [41,45-48]. Due to the ongoing debate about the suitability of lifestyles in comparison with demographics to explain media use patterns, our article provides new insight into this issue. Nevertheless, further studies are necessary to examine the influence of lifestyles beyond sociodemographic variables for different media use behaviors, especially among older people.

As a central part of health campaign planning, an adequate characterization of the addressees of a health message and a target group–specific implementation of the material often determines the success or failure of an intervention [73]. Our results showed that innovative digital health campaigns do not reach older adults as well as traditional media sources do. However, our study has shown that certain groups of older people can also be reached online; therefore, health information campaign planners should not only address sociodemographic variables but also general lifestyle types. Until now, only little was known about lifestyles in the age group 60 years and above except for a few studies such as one about preferences and perception of marketing communication of Poles aged 55 years and older [57] and a general lifestyle typology in the older American market [58]. Our study gives detailed insight into the lifestyles of older adults. This becomes relevant for health care communicators even outside older adults’ online health information–seeking behavior. Therefore, addressing the Sociable Adventurer with images of active older people may work better than images of inactive testimonials. Another conclusion could be drawn for the implementation of health campaign material: in order to address the Average Family Person, gardening or living magazines could be used for distribution. These potential differences should be tested in another study.

Additionally, studies have shown that older people prefer a wide range of content when using the internet, depending on their lifestyle [74,75]. It can be assumed that not only the general internet use differs between lifestyle types but also the way the internet is used: Sociable Adventurers are the most active and open-minded among the examined lifestyle types, which is why they may be more likely to use blogs or interactive offerings. Many people aged 60 years and older, however, are more family oriented. Therefore, they may use the internet primarily for interpersonal communication with their loved ones. If so, social media or mailings have the potential to reach this specific group. We recommend that future studies should investigate the explanatory potential of lifestyle types for the use of specific health services in the internet rather than just general internet use for health information.

### Limitations

This study has several limitations. First, we decided to focus on demographics and lifestyle types as defining factors of internet use for health information since other predictors such as trust, limited skills, or technophobia are defined as age-related [9,22]. However, the observed variables only explained 13% of the variance of older peoples’ online health information–seeking behavior. Future research should include other variables such as trust in the internet or technical skills to better explain older adults’ online health information–seeking behavior.

Further, the response rate was only 2.4%. Low response rates in telephone surveys can lead to biased data under the assumption that people who consistently participate in surveys are different than those who do not. This nonresponse bias occurs for civic and social engagement as well as volunteering [76]. Nevertheless, telephone polls show only minimal bias from nonresponses on lifestyle, health, and demographic questions, which leads us to the assumption that the nonresponse bias in our sample was low [76].

Last, the analyses are based on cross-sectional survey data. Therefore, we cannot make clear statements regarding causality and the sequence of relationships. Future research should focus on longitudinal data to investigate more support for the order of events.

### Conclusions

Our study focused on understanding the use of the internet for health information by adults 60 years and older and how lifestyle types can contribute to explain this behavior. Our study showed that older adults still use the internet very infrequently when seeking health information. However, a subgroup of older adults may be reached with online health information; this group includes the subset of younger, male senior citizens who like to spend their free time actively or with their family and enjoy working in their house and garden. Taking into account a predicted increase in internet use by the next generation of older adults, these individuals should be adequate recipients of online health information, while older inactive people should still be addressed with traditional media like health information brochures.

## Appendix

Multimedia Appendix 1 Descriptive analysis of all variables considered in the analysis.

Multimedia Appendix 2 Factor analysis of general values constructs.

Multimedia Appendix 3 Factor analysis of leisure activity constructs.

Multimedia Appendix 4 Factor analysis of interest constructs.

Multimedia Appendix 5 Descriptive analysis of the outcome variable for the three clusters.

Multimedia Appendix 6 Summary of hierarchical multiple regression analysis for variables predicting older adults’ online health information seeking behavior: changed reference group.

Multimedia Appendix 7 Summary of logistic regression analysis for variables predicting older adults’ online health information seeking behavior.

## Abbreviations

KMO: Kaiser-Meyer-Olkin
