# Supplementary material for: Lifestyle Segmentation to Explain the Online Health Information–Seeking Behavior of Older Adults: Representative Telephone Survey
Source: J Med Internet Res. 2020 Jun 12;22(6):e15099. doi: 10.2196/15099 (PMC7320311; doi:10.2196/15099)
Supplement: Multimedia Appendix 1 [file jmir_v22i6e15099_app1.docx]

Appendix 1. Descriptive analysis of all variables considered in the analysis.

|  | M | SD | N | Skewness  [SD] | Kurtosis  [SD] |
| --- | --- | --- | --- | --- | --- |
|  |  |  |  |  |  |
| **Sources of health information** |  |  |  |  |  |
| Health personnel | 2.89 | 1.11 | 689 | 0.03 [0.09] | -0.61 [0.19] |
| Family/friends | 2.88 | 1.03 | 689 | -0.15 [0.09] | -0.56 [0.19] |
| Brochures | 2.85 | 1.21 | 689 | -0.09 [0.09] | -0.97 [0.19] |
| Conventional media | 2.70 | 1.11 | 689 | 0.00 [0.09] | -0.80 [0.19] |
| Books | 2.02 | 1.10 | 686 | 0.79 [0.09] | -0.35 [0.19] |
| Pharmacy | 1.97 | 0.98 | 688 | 0.69 [0.09] | -0.39 [0.19] |
| Internet | 1.82 | 1.07 | 685 | 1.04 [0.09] | -0.10 [0.19] |
| Other patients | 1.80 | 0.94 | 682 | 0.94 [0.09] | 0.01 [0.19] |
| Health insurance | 1.42 | 0.71 | 682 | 1.87 [0.09] | 3.73 [0.19] |
| Health workshops | 1.29 | 0.71 | 681 | 2.61 [0.09] | 6.19 [0.19] |
| Apps | 1.12 | 0.47 | 684 | 4.56 [0.09] | 22.33 [0.19] |
|  |  |  |  |  |  |
| **Values & life goals** |  |  |  |  |  |
| Law and order | 4.68 | 0.59 | 686 | -1.93 [0.09] | 3.87 [0.19] |
| Safety | 4.37 | 0.83 | 685 | -1.46 [0.09] | 2.17 [0.19] |
| Success | 2.38 | 1.05 | 681 | 0.48 [0.09] | -0.34 [0.19] |
| Creativity | 3.80 | 1.08 | 684 | -0.89 [0.09] | 0.34 [0.19] |
| Political commitment | 2.69 | 1.33 | 684 | 0.28 [0.09] | -1.12 [0.19] |
| Amusement | 2.82 | 1.16 | 680 | 0.12 [0.09] | -0.83 [0.19] |
| Conformity | 1.79 | 0.94 | 678 | 1.14 [0.09] | 0.84 [0.19] |
| Friends | 4.52 | 0.76 | 683 | -1.88 [0.09] | 4.00 [0.19] |
| Environment | 4.37 | 0.74 | 684 | -1.45 [0.09] | 3.23 [0.19] |
| Peace and harmony | 4.58 | 0.62 | 686 | -1.62 [0.09] | 3.29 [0.19] |
| Religion | 2.72 | 1.44 | 673 | 0.13 [0.09] | -1.38 [0.19] |
|  |  |  |  |  |  |
| **Leisure values** |  |  |  |  |  |
| Experience adventures | 2.19 | 1.23 | 655 | 0.80 [0.10] | -0.45 [0.19] |
| Meet interesting people | 3.74 | 1.11 | 657 | -0.91 [0.10] | 0.26 [0.19] |
| Do sth. new/crazy | 2.24 | 1.30 | 654 | 0.71 [0.10] | -0.73 [0.19] |
|  |  |  |  |  |  |
| **Leisure activities** |  |  |  |  |  |
| Using a computer | 3.51 | 1.36 | 673 | -0.32 [0.10] | -1.25 [0.19] |
| Participating in sports | 3.12 | 1.36 | 673 | 0.07 [0.10] | -1.30 [0.19] |
| Going to the theatre/opera | 3.73 | 1.16 | 671 | -0.51 [0.10] | -0.71 [0.19] |
| Visiting a museum | 3.72 | 1.02 | 669 | -0.42 [0.10] | -0.56 [0.19] |
| Eating out in a restaurant | 3.47 | 0.95 | 673 | -0.20 [0.10] | -0.49 [0.19] |
| Having guests visit them at home | 2.90 | 0.90 | 674 | 0.38 [0.10] | -0.03 [0.19] |
| Reading books | 2.54 | 1.27 | 676 | 0.50 [0.10] | -0.83 [0.19] |
| Watching TV | 2.38 | 0.97 | 675 | 0.56 [0.10] | -0.09 [0.19] |
| Gardening | 2.85 | 1.41 | 677 | 0.37 [0.10] | -1.20 [0.19] |
| Doing handicraft work | 3.70 | 1.30 | 675 | -0.46 [0.10] | -1.16 [0.19] |
| Going for a walk | 2.72 | 1.17 | 675 | 0.51 [0.10] | -0.53 [0.19] |
| Baking/cooking | 2.63 | 1.50 | 675 | 0.49 [0.10] | -1.22 [0.19] |
| Listening to music | 2.26 | 1.09 | 676 | 0.76 [0.10] | -0.16 [0.19] |
| Spending time with their family | 2.39 | 1.19 | 676 | 0.82 [0.10] | -0.16 [0.19] |
|  |  |  |  |  |  |
| **Personal interests** |  |  |  |  |  |
| Politics | 4.24 | 1.05 | 672 | -1.42 [0.10] | 1.40 [0.19] |
| Economics/law | 3.64 | 1.25 | 672 | -0.57 [0.10] | -0.75 [0.19] |
| Sports | 3.36 | 1.26 | 671 | -0.32 [0.10] | -0.99 [0.19] |
| Health | 3.97 | 0.95 | 671 | -0.74 [0.10] | 0.13 [0.19] |
| Arts/literature | 3.33 | 1.19 | 670 | -0.16 [0.10] | -0.96 [0.19] |
| Science | 3.44 | 1.18 | 670 | -0.36 [0.10] | -0.78 [0.19] |
| Children/upbringing | 3.42 | 1.40 | 666 | -0.47 [0.10] | -1.08 [0.19] |
| Partnership/family | 4.38 | 0.99 | 667 | -1.90 [0.10] | 3.21 [0.19] |
| Travelling/vacation | 3.55 | 1.22 | 669 | -0.48 [0.10] | -0.74 [0.19] |
| Work | 2.87 | 1.51 | 661 | 0.11 [0.10] | -1.46 [0.19] |
| House/garden | 3.78 | 1.26 | 668 | -0.88 [0.10] | -0.28 [0.19] |
|  |  |  |  |  |  |
| **All EFA dimensions (z-stand.)** |  |  |  |  |  |
| Regularity | 0.00 | 0.81 | 681 | -1.57 [0.09] | 2.67 [0.19] |
| Harmony | 0.00 | 0.60 | 681 | -1.42 [0.09] | 2.67 [0.19] |
| Culture | 0.00 | 0.87 | 668 | 0.38 [0.10] | -0.58 [0.19] |
| Technology | 0.00 | 0.62 | 668 | 0.16 [0.10] | -0.12 [0.19] |
| Home and garden | 0.00 | 0.78 | 668 | -0.01 [0.10] | -0.91 [0.19] |
| News | 0.00 | 0.83 | 658 | -0.92 [0.10] | 0.36 [0.19] |
| Family | 0.00 | 0.86 | 658 | -0.51 [0.10] | -0.74 [0.19] |
