# Supplementary material for: Lifestyle Segmentation to Explain the Online Health Information–Seeking Behavior of Older Adults: Representative Telephone Survey
Source: J Med Internet Res. 2020 Jun 12;22(6):e15099. doi: 10.2196/15099 (PMC7320311; doi:10.2196/15099)
Supplement: Multimedia Appendix 2 [file jmir_v22i6e15099_app2.docx]

| Appendix 2. Factor analysis of general values constructs. | | |
| --- | --- | --- |
|  | **Factor loading** | |
| **Item** | **1** | **2** |
|  |  |  |
| **Factor 1: Regularity (r = .51)** |  |  |
|  |  |  |
| Security | .71 | .13 |
| Law and order | .70 | .09 |
|  |  |  |
| **Factor 2: Harmony (r = .23)** |  |  |
|  |  |  |
| Friends | .12 | .48 |
| Peace/harmony | .04 | .46 |
|  |  |  |
| Eigenvalues | 1.63 | 1.11 |
| Percent of variance explained | 40.76 | 27.85 |
|  |  |  |
