# Supplementary material for: Lifestyle Segmentation to Explain the Online Health Information–Seeking Behavior of Older Adults: Representative Telephone Survey
Source: J Med Internet Res. 2020 Jun 12;22(6):e15099. doi: 10.2196/15099 (PMC7320311; doi:10.2196/15099)
Supplement: Multimedia Appendix 3 [file jmir_v22i6e15099_app3.docx]

| Appendix 3. Factor analysis of leisure activity constructs. | | |  |
| --- | --- | --- | --- |
|  | **Factor loading** | | |
| **Item** | **1** | **2** | **3** |
|  |  |  |  |
| **Factor 1: Culture (r = .57)** |  |  |  |
|  | | |  |
| Going to a theatre/opera | .80 | .08 | -.12 |
| Visiting museums | .73 | .03 | .12 |
|  |  |  |  |
| **Factor 2: Home and garden** **(r = .35)** |  |  |  |
|  |  |  |  |
| Handicraft work | .04 | .64 | .27 |
| Gardening | .07 | .64 | -.21 |
|  |  |  |  |
| **Factor 3: Technology (r = -.12)** |  |  |  |
|  |  |  |  |
| Baking/cooking | .13 | .14 | .40 |
| Using a computer | .08 | .09 | -.35 |
|  |  |  |  |
| Eigenvalues | 1.72 | 1.29 | 1.19 |
| Percent of variance explained | 28.64 | 21.48 | 19.68 |
|  |  |  |  |
