# Supplementary material for: Lifestyle Segmentation to Explain the Online Health Information–Seeking Behavior of Older Adults: Representative Telephone Survey
Source: J Med Internet Res. 2020 Jun 12;22(6):e15099. doi: 10.2196/15099 (PMC7320311; doi:10.2196/15099)
Supplement: Multimedia Appendix 4 [file jmir_v22i6e15099_app4.docx]

| Appendix 4. Factor analysis of interest constructs. | | |
| --- | --- | --- |
|  | **Factor loadings** | |
| **Item** | **1** | **2** |
|  |  |  |
| **Factor 1: News (r = .53)** |  |  |
|  |  |  |
| Economics/law | .10 | .72 |
| Politics | .08 | .72 |
|  |  |  |
| **Factor 2: Family (α = .63)** |  |  |
|  |  |  |
| Children/upbringing | .82 | .01 |
| Partnership/family | .52 | .10 |
| Work/education | .48 | .09 |
|  |  |  |
| Eigenvalues | 1.90 | 1.36 |
| Percent of variance explained | 37.95 | 27.23 |
|  |  |  |
