# Supplementary material for: Lifestyle Segmentation to Explain the Online Health Information–Seeking Behavior of Older Adults: Representative Telephone Survey
Source: J Med Internet Res. 2020 Jun 12;22(6):e15099. doi: 10.2196/15099 (PMC7320311; doi:10.2196/15099)
Supplement: Multimedia Appendix 5 [file jmir_v22i6e15099_app5.docx]

Appendix 5. Descriptive analysis of the outcome variable for the three clusters.

|  | The Sociable Adventurer (n=169) | | | The Average Family Person (n=306) | | | The Uninterested Inactive (n=117) | | |
| --- | --- | --- | --- | --- | --- | --- | --- | --- | --- |
|  | M [SD] | Skew-ness [SD] | Kur-tosis [SD] | M [SD] | Skew-ness [SD] | Kur-tosis [SD] | M [SD] | Skew-ness [SD] | Kur-tosis [SD] |
|  |  |  |  |  |  |  |  |  |  |
| Internet use for health information | 2.11 [1.14] | 0.64 [0.19] | -0.75 [0.37] | 1.85 [1.08] | 0.97 [0.14] | -0.21 [0.28] | 1.42 [0.77] | 2.06 [0.22] | 4.48 [0.44] |
|  |  |  |  |  |  |  |  |  |  |
