# Supplementary material for: Lifestyle Segmentation to Explain the Online Health Information–Seeking Behavior of Older Adults: Representative Telephone Survey
Source: J Med Internet Res. 2020 Jun 12;22(6):e15099. doi: 10.2196/15099 (PMC7320311; doi:10.2196/15099)
Supplement: Multimedia Appendix 6 [file jmir_v22i6e15099_app6.docx]

Appendix 6. Summary of hierarchical multiple regression analysis for variables predicting older adults’ online health information seeking behavior; changed reference group ^b^

|  | Step 1 | | | | Step 2 | | | |
| --- | --- | --- | --- | --- | --- | --- | --- | --- |
|  | B | SE B | β | P | B | SE B | β | P |
|  |  |  |  |  |  |  |  |  |
| **Step 1** |  |  |  |  |  |  |  |  |
|  |  |  |  |  |  |  |  |  |
| Age | -.033 | .005 | -.236 | <.001 | -.029 | .006 | -.208 | <.001 |
| Gender | -.281 | .085 | -.131 | .001 | -.323 | .085 | -.151 | <.001 |
| Education | .270 | .087 | .126 | .002 | .199 | .088 | .093 | .025 |
| Place of residence | .184 | .096 | .076 | .054 | .155 | .096 | .064 | .11 |
| Health status | .102 | .050 | .081 | .041 | .068 | .051 | .054 | .18 |
|  |  |  |  |  |  |  |  |  |
| **Step 2** |  |  |  |  |  |  |  |  |
|  |  |  |  |  |  |  |  |  |
| The Sociable Adventurer (Dummy) | - | - | - | - | .098 | .101 | .042 | .33 |
| The Uninterested Inactive (Dummy) | - | - | - | - | -.341 | .114 | -.127 | .003 |
|  |  |  |  |  |  |  |  |  |

*^b^ N* = 587; weighted sample; *R^2^_Step1_* = .114, *R^2^_Step2_* = .132.
