# Supplementary material for: Lifestyle Segmentation to Explain the Online Health Information–Seeking Behavior of Older Adults: Representative Telephone Survey
Source: J Med Internet Res. 2020 Jun 12;22(6):e15099. doi: 10.2196/15099 (PMC7320311; doi:10.2196/15099)
Supplement: Multimedia Appendix 7 [file jmir_v22i6e15099_app7.docx]

Appendix 7. Summary of logistic regression analysis for variables predicting older adults’ online health information seeking behavior ^b^

|  | Step 1 | | | | Step 2 | | | |
| --- | --- | --- | --- | --- | --- | --- | --- | --- |
|  | B | SE B | Exp(b) | P | B | SE B | Exp(b) | P |
|  |  |  |  |  |  |  |  |  |
| **Step 1** |  |  |  |  |  |  |  |  |
|  |  |  |  |  |  |  |  |  |
| Age | -.078 | .012 | 0.925 | <.001 | -.073 | .013 | 0.930 | <.001 |
| Gender | .593 | .180 | 1.810 | .001 | .689 | .185 | 1.992 | <.001 |
| Education | -.602 | .184 | 0.548 | .001 | -.468 | .190 | 0.626 | .014 |
| Place of residence | -.190 | .203 | 0.827 | .351 | -.121 | .208 | 0.886 | .559 |
| Health status | .266 | .107 | 1.304 | .013 | .201 | .111 | 1.222 | .070 |
|  |  |  |  |  |  |  |  |  |
| **Step 2** |  |  |  |  |  |  |  |  |
|  |  |  |  |  |  |  |  |  |
| The Sociable Adventurer (Dummy) | - | - | - | - | -.891 | .291 | 0.410 | .002 |
| The Average Family Person (Dummy) | - | - | - | - | -.635 | .257 | 0.530 | .013 |
|  |  |  |  |  |  |  |  |  |

*^b^ N* = 580; weighted sample; *Nagelkerkes R^2^_Step1_* = .169, *Nagelkerkes R^2^_Step2_* = .188.
